# The epidemiology of fracture-related infections in Germany

**DOI:** 10.1038/s41598-021-90008-w

**Published:** 2021-05-17

**Authors:** Nike Walter, Markus Rupp, Siegmund Lang, Volker Alt

**Affiliations:** 1 Department for Trauma Surgery, University Medical Center Regensburg, Franz-Josef-Strauß-Allee 11, 93053 Regensburg, Germany; 2 Department for Psychosomatic Medicine, University Medical Center Regensburg, Regensburg, Germany

**Keywords:** Health care, Medical research

## Abstract

The epidemiology of fracture-related infection (FRI) is unknown, which makes it difficult to estimate future demands and evaluate progress in infection prevention. Therefore, we aimed to determine the nationwide burden’s development over the last decade as a function of age group and gender. FRI prevalence as a function of age group and gender was quantified based on annual ICD-10 diagnosis codes from German medical institutions between 2008 through 2018, provided by the Federal Statistical Office of Germany (Destatis). The prevalence of FRI increased by 0.28 from 8.4 cases per 100,000 inhabitants to 10.7 cases per 100,000 inhabitants between 2008 and 2018. The proportion of fractures resulting in FRI increased from 1.05 to 1.23%. Gender distribution was equal. Patients aged 60–69 years and 70–79 years comprised the largest internal proportion with 20.2% and 20.7%, respectively, whereby prevalence increased with age group. A trend towards more diagnoses in older patients was observed with a growth rate of 0.63 for patients older than 90 years. Increasing rates of fracture-related infection especially in older patients indicate an upcoming challenge for stakeholders in health care systems. Newly emerging treatment strategies, prevention methods and interdisciplinary approaches are strongly required.

## Introduction

In trauma surgery, reduction and internal fixation is applied to restore skeletal integrity. One of the major complications after fracture fixation utilizing metallic fracture fixation devices, is implant related infection, which in general requires surgical treatment Depending on several factors, at least one, but often two or even multiple staged surgeries are needed for eradication of infection and finally bony consolidation^1^. In the literature, rates of developing a posttraumatic infection are reported to be around 1–2% for closed fractures ranging up to exceeding 30% for Gustilo-Anderson type III open tibia fractures^2–4^. Considering studies showing that incidences of long bone fractures increase^5^, numbers of infection complications can be expected to rise as well.

Depending on injury severity, success rates only vary between 70–90% with a recurrence of the infection in 6–9% of the patients^1,6,7^. Among others, consequences are significantly reduced patient-reported quality of life and multiplied healthcare costs up to 6.5 times^8,9^. Hence, much effort has been made in prevention approaches^2,10–12^.

However, current socioeconomic calculations are based on small patient numbers and the exact prevalence of fracture-related infection is unknown. Therefore, it remains difficult to estimate future demands foresee developments and evaluate the progress in infection prevention methods. To this end, we aimed at determining the nationwide burden and analysing recent trends in fracture-related infections.

## Material and methods

Data consisting of annual ICD-10 diagnosis codes from German medical institutions between 2008 through 2018 was provided by the Federal Statistical Office of Germany (Destatis). The ICD-10 code “T84.6, infection and inflammatory reaction due to internal fixation device” was used to identify patients aged 20 years or older diagnosed with FRI. A detailed breakdown of these data by age group and gender was performed. Prevalence rates were calculated based on Germany’s historical population aged 20 years or older provided by Destatis^13^. Here, the number of inhabitants in each of the 16 German federal states was considered by year of birth for each year of the period 2008 through 2018. The deadline of each year was December 31. The proportion of FRI was calculated based on total numbers of fracture diagnoses. Here, the ICD-10 codes shown in Table 1 were used (Table 1). Data were analyzed using the statistical software SPSS Version 26.0 (IBM, SPSS Inc. Armonk, NY, USA).

**Table 1:** Used ICD-10 code to calculate total numbers of fractures with descriptions.

ICD-10 code	Description	ICD-10 code	Description
S32.1	Sacrum fracture	S72.0	Femur neck fracture
S32.2	Coccyx fracture	S72.1	Pertrochanteric femur fracture
S32.3	Ilium fracture	S72.2	Subtrochanteric femur fracture
S32.4	Acetabulum fracture	S72.3	Femur shaft fracture
S32.5	Pubis fracture	S72.4	Distal femur fracture
S32.6	Ischium fracture	S82.0	Patella fracture
S32.8	Fracture of other parts of pelvis	S82.1	Proximal tibia fracture
S42.0	Clavicle fracture	S82.2	Tibia shaft fracture
S42.1	Scapula fracture	S82.3	Distal tibia fracture
S42.2	Proximal humerus fracture	S82.4	Fibula shaft fracture
S42.3	Humerus shaft fracture	S82.5	Medial malleolus fracture
S42.4	Distal humerus fracture	S82.6	Lateral malleolus fracture
S52.0	Proximal ulna fracture	S92.0	Calcaneus fracture
S52.1	Proximal radius fracture	S92.1	Talus fracture
S52.2	Ulna shaft fracture	S92.2	Other tarsal bone(s) fracture
S52.3	Radius shaft fracture	S92.3	Metatarsal bone(s) fracture
S52.5	Distal radius fracture		
S62.0	Scaphoid fracture		
S62.1	Carpal bone fracture		
S62.2	First metacarpal bone fracture		
S62.3	Other metacarpal bones fracture		

## Results

In 2018, a total number of 7253 FRI cases were listed in Germany. In comparison to 5556 cases in 2008, the overall prevalence substantially increased with a growth rate of 0.28 from 8.4 cases per 100,000 inhabitants to 10.7 cases per 100,000 inhabitants. Accordingly, the proportion of fractures resulting in FRI increased from 1.04 to 1.23% (Table 2).

**Table 2:** Historic development of population and fracture-related infection prevalence from 2008 through 2018.

Year	Total numbers	German population 20 years or older	Prevalence per 100,000 inhabitants	Growth rate (relative to 2008)	Fractures total numbers	Proportion of FRI
2008	5556	66,346,045	8.4	–	534,131	1.04
2009	6091	66,400,066	9.2	0.10	578,897	1.05
2010	6503	66,549,975	9.8	0.17	553,012	1.18
2011	6800	65,398,514	10.4	0.24	547,319	1.24
2012	6735	65,665,069	10.3	0.22	556,766	1.21
2013	6985	65,943,867	10.6	0.26	547,683	1.28
2014	6882	66,677,665	10.3	0.23	562,294	1.22
2015	7206	67,097,676	10.7	0.28	568,598	1.27
2016	7024	67,440,230	10.4	0.24	580,975	1.21
2017	7228	67,540,025	10.7	0.28	585,891	1.23
2018	7253	67,724,921	10.7	0.28	587,612	1.23

**Figure 1:**
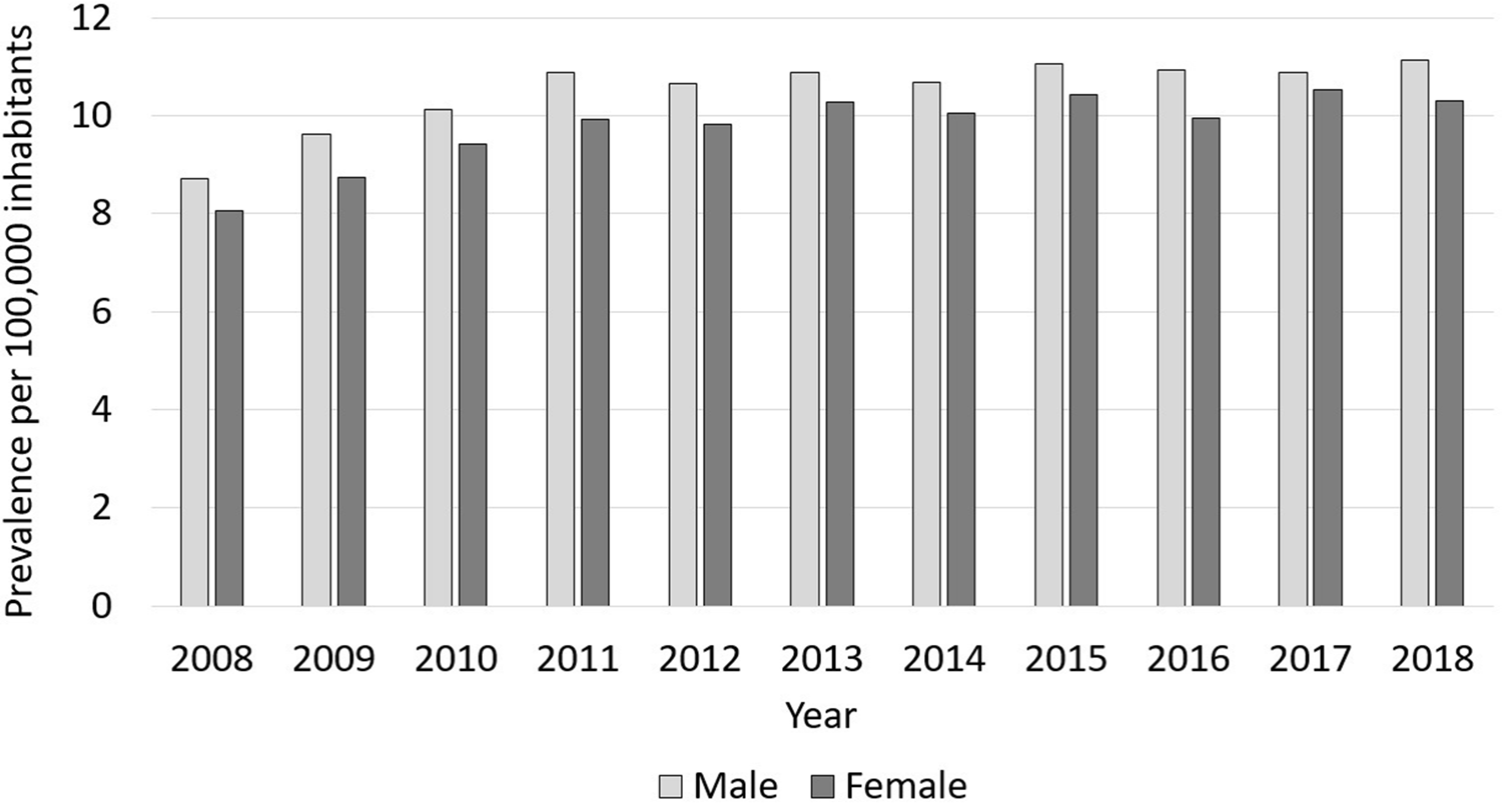
Development of FRI prevalence from 2008 to 2018. The prevalence of men diagnosed with FRI is shown in light grey, the prevalence of female cases is illustrated in dark grey.

The internal gender distribution was equal with 50.8% male cases and 49.2% cases in 2018, whereby the prevalence of FRI was slightly higher in the male population (11.1 cases per 100,000 inhabitants) than for the female population (10.3 cases per 100,000 inhabitants) (Fig. 1, Table 3).

**Table 3:** Historic development from 2008 through 2018 of all fracture-related infection cases as a function of gender.

Year	Male cases			Female cases		
	Total numbers (percentage)	Prevalence per 100,000 male inhabitants	Growth rate (relative to 2008)	Total number (percentage)	Prevalence per 100,000 female inhabitants	Growth rate (relative to 2008)
2008	2802 (50.4)	8.7	–	2754 (49.6)	8.0	–
2009	3104 (51.0)	9.6	0.11	2987 (49.0)	8.7	0.08
2010	3275 (50.4)	10.1	0.16	3228 (49.6)	9.4	0.17
2011	3440 (50.6)	10.9	0.25	3360 (49.4)	9.9	0.23
2012	3394 (50.4)	10.7	0.22	3341 (49.6)	9.8	0.22
2013	3482 (49.8)	10.9	0.25	3503 (50.2)	10.3	0.28
2014	3447 (50.1)	10.7	0.23	3435 (49.9)	10.0	0.25
2015	3619 (50.2)	11.1	0.27	3587 (49.8)	10.4	0.30
2016	3591 (51.1)	10.9	0.26	3433 (48.9)	10.0	0.24
2017	3586 (49.6),	10.9	0.25	3642 (50.4)	10.5	0.31
2018	3682 (50.8)	11.1	0.28	3571 (49.2)	10.3	0.28

**Figure 2:**
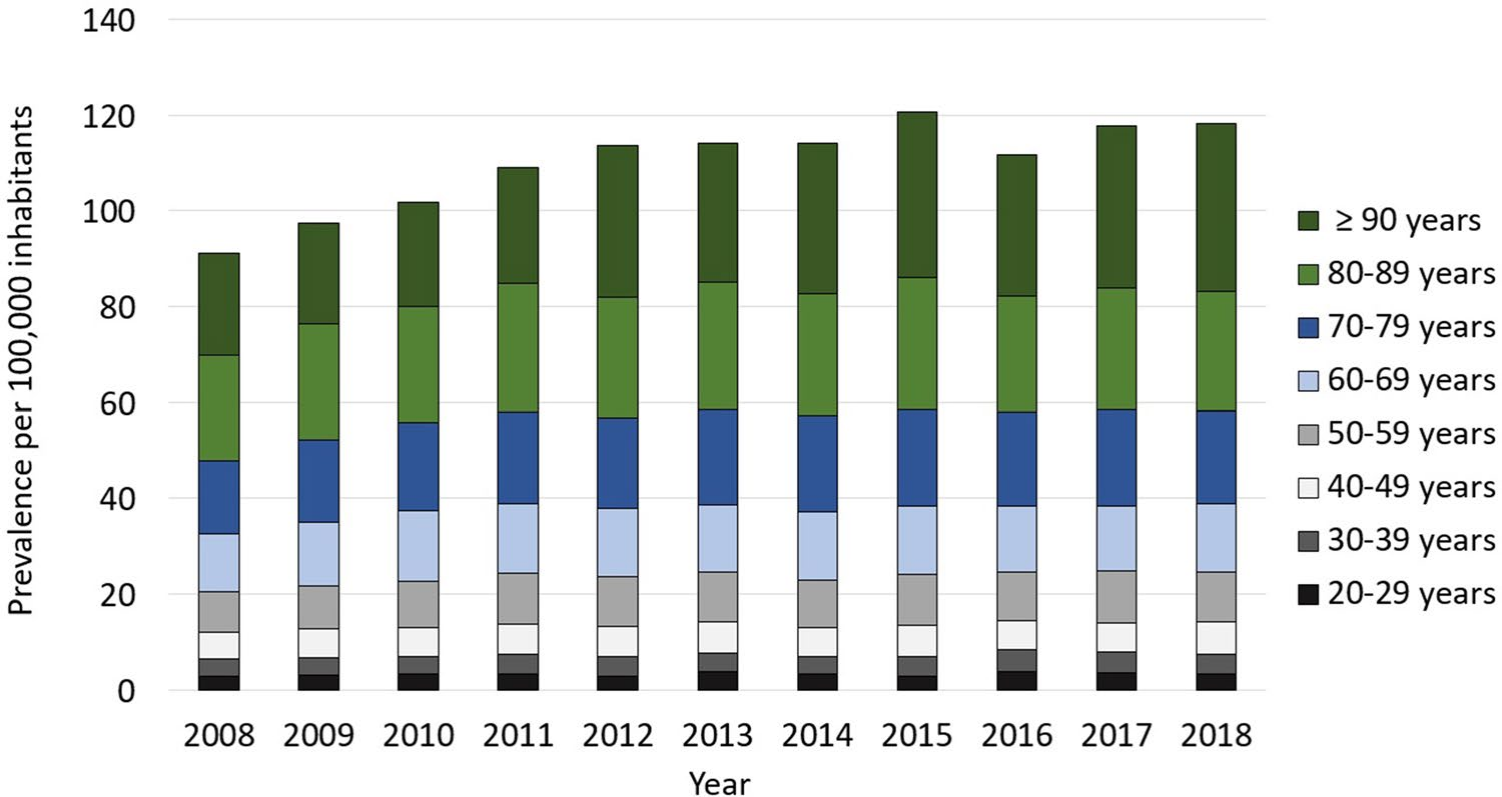
Development of FRI prevalence from 2008 to 2018 as a function of age group in 10-year increments.

Regarding the prevalence for distinct age groups, cases per 100,000 inhabitants steadily increased with age. For 2018, 35 cases were calculated per 100,000 per inhabitants aged 90 years or older, 24.9 cases per 100,000 per inhabitants aged 80–89 years and 19.5 cases per 100,000 per inhabitants aged 70–79 years, whereas only 3.4 cases were estimated per 100,000 per inhabitants aged 20–29 years and 4.1 cases per 100,000 per inhabitants aged 30–39 years. Relative to the year 2008, a trend towards more FRI diagnoses in older patients can be observed. Highest growth rates were found for patients aged 90 years or older (0.63) and patients aged 70–79 years (0.28) (Table 4, Fig. 2).

**Table 4:** Historic development from 2008 through 2018 of all fracture-related infection cases as a function of age group. Data is shown as total numbers, percentage and prevalence per 100,000 inhabitants of the considered age group.

Year	20–29 years Total (percen-tage), preva-lence	30–39 years Total (percen-tage), preva-lence	40–49 years Total (percen-tage), preva-lence	50–59 years Total (percen-tage), preva-lence	60–69 years Total (percen-tage), preva-lence	70–79 years Total (percen-tage), preva-lence	80–89 years Total (percen-tage), preva-lence	90 years or older Total (percen-tage), preva-lence
2008	274 (4.9), 2.8	393 (7.1), 3.8	753 (13.6), 5.4	969 (17.4), 8.6	1136 (20.4), 12.1	1146 (20.6), 15.2	756 (13.6), 21.8	129 (2.3), 21.5
2009	319 (5.2), 3.2	363 (6.0), 3.6	821 (13.5), 5.9	1035 (17.0), 9.0	1208 (19.8), 13.1	1355 (22.2), 17.3	859 (14.1), 24.1	131 (2.2), 21.0
2010	324 (5.0), 3.3	368 (5.7), 3.8	827 (12.7), 6.0	1137 (17.5), 9.7	1326 (20.4), 14.7	1491 (22.9), 18.3	888 (13.7), 24.3	142 (2.2), 21.8
2011	321 (4.7), 3.3	385 (5.7), 4.1	836 (12.3), 6.3	1251 (18.4), 10.6	1291 (19.0), 14.6	1587 (23.3), 19.0	973 (14.3), 26.8	156 (2.3), 24.2
2012	283 (4.2), 2.9	384 (5.7), 4.0	823 (12.2), ), 6.4	1244 (18.5), 10.3	1271 (18.9), 14.2	1593 (23.7), 18.9	926 (13.7), 25.3	211 (3.1), 31.7
2013	378 (5.4), 3.9	373 (5.3), 3.9	804 (11.5), 6.5	1284 (18.4), 10.4	1259 (18.0), 14.0	1716 (24.6), 20.0	971 (13.9), 26.4	200 (2.9), 29.0
2014	322 (4.7), 3.3	360 (5.2), 3.7	710 (10.3), 6.0	1285 (18.7), 10.1	1289 (18.7), 14.1	1715 (24.9), 20.1	975 (14.2), 25.5	226 (3.3), 31.6
2015	292 (4.1), 2.9	422 (5.9), 4.2	735 (10.2), 6.4	1382 (19.2), 10.6	1370 (19.0), 14.4	1647 (22.9), 20.0	1110 (15.4), 27.7	248 (3.4), 34.5
2016	371 (5.3), 3.7	474 (6.7), 4.6	686 (9.8) , 6.2	1320 (18.8), 10.0	1361 (19.4), 13.8	1579 (22.5), 19.7	11,012 (14.4), 24.1	221 (3.1), 29.5
2017	345 (4.8), 3.5	459 (6.4), 4.4	659 (9.1 , 6.1	1438 (19.9), 10.8	1370 (19.0), 13.6	1588 (22.0), 20.2	1114 (15.4), 25.3	255 (3.5), 33.8
2018	333 (4.6), 3.4	435 (6.0), 4.1	701 (9.7), 6.7	1399 (19.3), 10.4	1463 (20.2), 14.2	1502 (20.7), 19.5	1154 (15.9) , 24.9	266 (3.7), 35.0
Growth rate (2018 relative to 2008)	0.23	0.07	0.25	0.21	0.17	0.28	0.14	0.63

**Figure 3:**
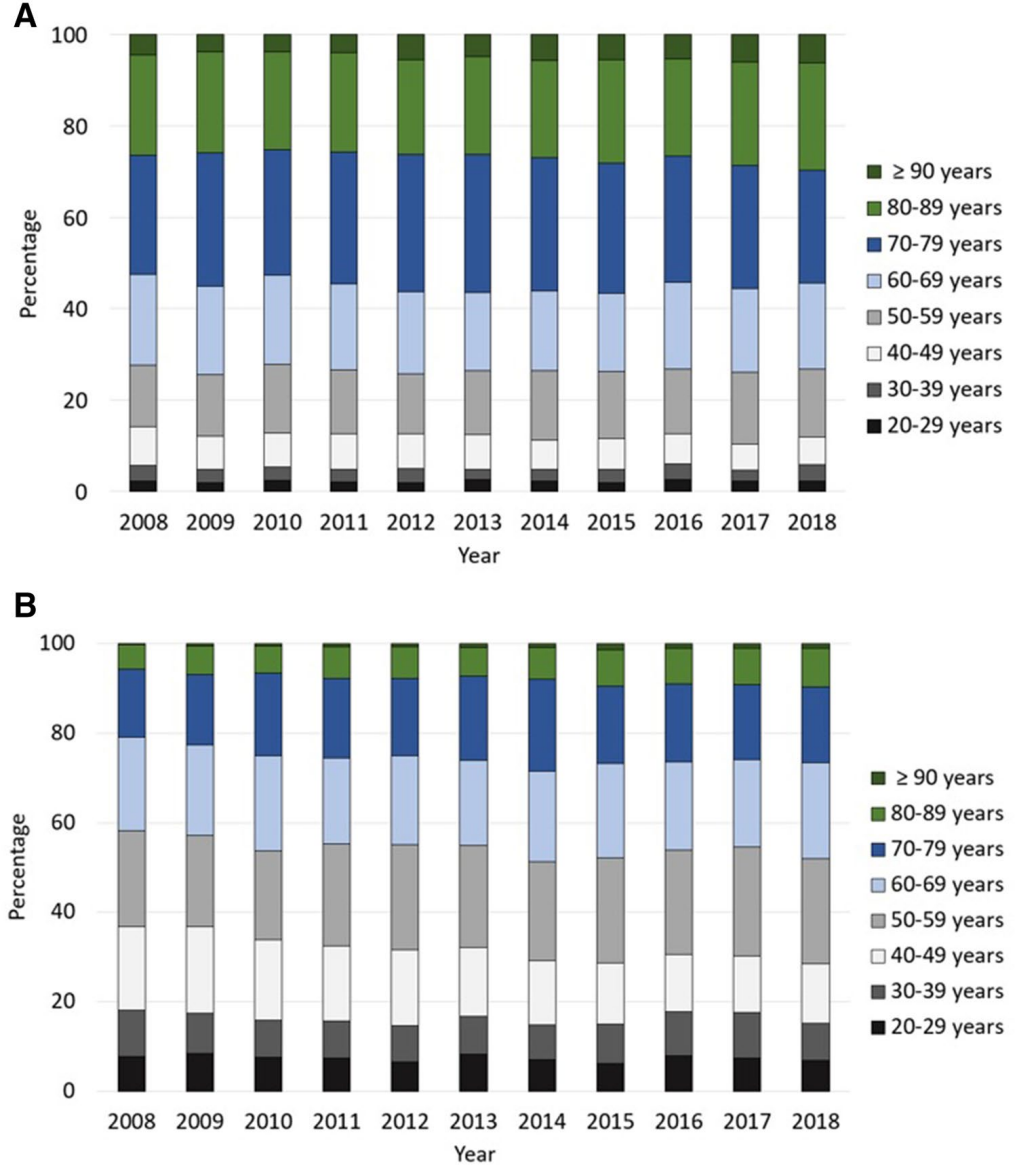
(**A**) Development of the internal proportion of male FRI cases divided by age group. (**B**) Development of the internal proportion of female FRI cases divided by age group.

Regarding the constituent ratio, explaining the internal proportion of infection, in 2018 patients aged 70–79 years comprised the largest cohort with 20.7%, followed by patients aged 60–69 years (20.2%) and patients aged 50–59 years (19.3%). Comparing the age distribution as a function of gender, it becomes apparent that older patients were predominantly female. For instance, 6.30% of female cases were aged 90 years or older compared to 1.11% male cases, 23.47% female patients aged 80–89 years versus 8.58% male cases in this increment and 24.64% women aged 70–79 years in relation to 16.89% men of this age. In the age increments 50–59 years, more male cases were registered than female cases (23.60% versus 14.84%). The same applied for patients aged 40–49 years with 13.17% male cases compared to 6.05% affected women (Fig. 3).

## Discussion

In this population-based study, trends in the epidemiology of fracture-related infections were described and prevalence was analyzed as a function of gender and age group. To the best of our knowledge, this study is the first one describing the nationwide burden of FRI.

A literature review estimated that fracture-fixation device infections comprise < 5% of all implant associated infections^14^, whereas a single center cohort study at Geneva University Hospital pooling clinical data on orthopaedic infections reported that 24% of all cases involved osteosynthetic material^15^. In general, prevalence data on FRI vary in the literature. For instance, a multi-center study carried out in India included 787 participants with tibia fractures, estimating the incidence of infection as 1.6% for closed fractures and 8.0% for open fractures^16^, whereas Metsemakers and colleagues found an infection rate of 3.4% in a cohort of 358 patients with tibia fractures^9^. Blonna et al. reported an infection rate of 4% out of 452 proximal humeral fractures and Ovaska et al. identified 5% of 1923 consecutive ankle fractures to be infected^17,18^. One study carried out in Brazil found an infection rate of 13.24%, examining 142 patients with open fractures at various anatomical locations, whereas another one reported 18.8% infections in 133 patients with open fractures^19,20^. Additionally, a review on open femoral shaft fractures treated with intramedullary nailing estimated an infection rate of 6%, whereas another review reported infection rates in the range of 0.9–11.6% comparing outcomes of open tibial fractures^21,22^. In light of the diversity of findings, describing the nationwide burden of FRI seems useful. Here, an overall FRI rate of 1.23% was estimated for the year 2018 based on calculated total numbers of fracture cases, which is lower than previously reported. Differences might be explainable by the distinct considered fracture types and sites, heterogeneity in the study design as well as center-specific treatment procedures.

Further, our analysis revealed that the prevalence of FRI increased with a rate of 0.28 from 8.4 cases per 100,000 inhabitants to 10.7 cases per 100,000 inhabitants between 2008 and 2018. The distribution of male and female cases was equal in our analysis, whereas research increasingly addresses immune response gender differences^23,24^. The observed trend towards more FRI diagnosis in older patients possibly reflects demographic changes such as population decline and aging, which challenge the healthcare system not only in Germany. In consideration that prevention strategies and improved treatment algorithms for optimal patient care moved into focus of orthopedic research^2,10,25^, the increase of infection rates over the last decade seems surprisingly high. This might be attributable to heightened prevalence of obesity, which has risen substantially and the fact that Germany is rated among the countries with the highest prevalence of tobacco use in Europe^26,27^. Further, an extrapolation of hospital-based data to the German population revealed 16,742 severely injured persons per year and at least 5.8 million German inhabitants have received a medical diagnosis of type 2 diabetes, which may contribute to rising FRI numbers^28–30^.

Our study shows several limitations. First, the ICD-10 codes do not allow a differentiation regarding anatomical localization, classification of fractures as well as surgical treatment strategies. Further, it was not possible to derive individual features of the patients and risk factors such as obesity, smoking and comorbidities. Also, no statement about underlying pathogens causing the infection can be made. Finally, analyzing large registry data does not allow to apply in every treated case the FRI diagnosis criteria recently described by a consensus group^31^. This downside goes hand in hand with the upside of analyzing a complete data set, since all patients treated for FRI, which is in general an inpatient procedure have been coded by the OPS-code T84.6.

In conclusion, in light of a strong increase especially in elderly patients, prevention strategies, improved treatment strategies and an interdisciplinary treatment approaches are strongly required.

## Data Availability

The datasets analysed during the current study are available from the corresponding author on reasonable request.
